# Synthesis and Photoluminescence Properties of Pr^3+^-Doped Ba_0.5_Ca_0.5_Ti_x_Zr_(1-x)_O_3_ Perovskite Diphasic Ceramics Obtained by the Modified Pechini Method

**DOI:** 10.3390/ma15031058

**Published:** 2022-01-29

**Authors:** Agnieszka Wilk, Lucjan Kozielski, Daniel Michalik, Dawid Kozień, Jolanta Makowska, Zbigniew Pędzich

**Affiliations:** 1 Department of Ceramics and Refractories, Faculty of Materials Science and Ceramics, AGH-University of Science and Technology, 30 Mickiewicza Avenue, 30-059 Krakow, Poland; kozien@agh.edu.pl (D.K.); pedzich@agh.edu.pl (Z.P.); 2 Faculty of Science and Technology, University of Silesia, 1A 75 Pułku Piechoty St., 41-500 Chorzów, Poland; lucjan.kozielski@us.edu.pl; 3 Faculty of Materials Engineering, Silesian University of Technology, 8 Krasińskiego St., 40-019 Katowice, Poland; dmpolsl@googlemail.com

**Keywords:** photoluminescence, perovskite crystal structure, rare-earth-element doping, carrier trap, dielectric properties

## Abstract

The Pr^3+^-doped solid solutions from (Ba,Ca)(Ti,Zr)O_3_ (BCTZO) system were successfully synthesized using an efficient and low-energy consuming route—the Pechini method combined with the sintering at relatively low temperature (1450 °C). The obtained materials were characterized by means of X-ray diffraction (XRD) and scanning electron microscopy (SEM). The dielectric properties were systematically studied. The Pr^3+^-doped BCTZO diphasic material generates intense and broad red photoluminescence (PL) emission at room temperature. The optical properties were significantly improved with the Ti^4+^ substitution by Zr^4+^ ions. As a result, the Pr^3+^-doped (Ba,Ca)(Ti,Zr)O_3_ ceramics is a promising candidate for environmentally friendly, multifunctional material by combining good dielectric and photoluminescent properties with prognosis for the manifestation of strong photoluminescent and mechanoluminescent effects.

## 1. Introduction

Rapid and intense development in the field of electromechanical devices directed the interest of the researchers toward multifunctional materials, which possess two or more desirable characteristics realized by the entity [1,2,3]. Unique, two-parameter coupling effect and remarkable electrical and optical properties could be obtained in the Pr^3+^-doped Ba-Ca-Ti-Zr-O system. This presented research reports on multifunctional materials series exhibiting stable dielectric properties in addition to a strong photoluminescent effect.

The luminescence phenomenon is manifested by the emission of energy in form of light, intrinsically related to the release of charge carriers from the traps. Traps are defined as all kinds of dopants, impurities, vacancies, and other defects of the crystal structure, that provide additional unfilled energy levels into the bandgap [2]. Moreover, it has been confirmed that the presence of a dopant modifies the position of other traps in the energy diagram and stabilizes vacancies through electrostatic interaction [3]. The phosphors are called lanthanide-activated luminescent materials that are a combination of host lattice and a very low concentration of activator ions. It was established that the addition of rare earth elements (RE) that have their own intrinsic luminescent characteristics, which are added to those of the host, enhances the photoluminescence properties of the perovskite materials and help to obtain the desired emission effect [2].

Barium titanate (BaTiO_3_) is a promising lead-free material, which possesses several multifunctional properties. The excellent dielectric, piezoelectric, ferroelectric, and luminescent features of this titanate are well known [4]. In 2009, Liu and Ren [5] reported, for the first time, a very high piezoelectric coefficient of 620 pC/N at a morphotropic phase boundary for the Ba-Ca-Ti-Zr-O system. Consequently, the Ba-Ca-Ti-Zr-O ceramics gained a tremendous amount of research interest in recent studies. In order to improve electrical and optical properties, considerable attention is directed at Ca^2+^ and Zr^4+^ modification of the BaTiO_3_ structure [6]. Zirconium ions (0.72 Å), which tend to replace titanium ones (0.64 Å), are chemically more stable, enable the control of the temperature stability, decrease the Curie temperature (T_C_), and increase the orthorhombic–tetragonal phase transition temperature (T_O-T_) [7,8]. Moreover, Zr^4+^ in B sites changes the nature of the phase transition from normal to relaxor ferroelectric behavior [9]. When it comes to optical properties, Zr^4+^ addition could significantly enhance the red fluorescence intensity [10]. Intense and broad photoluminescence emission was observed in structurally disordered Ba(Ti,Zr)O_3_ and Ca(Ti,Zr)O_3_. Cavalcante et al. [11,12] linked the intense and broad photoluminescence directly with an order–disorder degree in the lattice and created a wideband model based on the electronic transitions between localized intermediate energy levels in the bandgap formed mainly by the structural defects. Moreover, local structure deformations lead to a charge discontinuity. This is in agreement with the results of Parida et al. [13] and Anicete-Santos et al. [14], who linked the photoluminescence (PL) behavior with the existence of charge gradient on the disordered structure and possible charge transfer process between [TiO_x_]-[ZrO_x_] clusters.

Calcium ions (1.06 Å) tend to substitute the barium ones (1.43 Å) in Ba_y_Ca_1-y_TiO_3_ solid solutions, which relates to its ionic radius. The T_C_ value remains almost constant, while T_R-O_ and T_O-T_ transition temperatures are markedly lowered [15]. It has been known that Ca^2+^ ions substitute the Ba^2+^ ions to form an insolubility region when the amount of Ca^2+^ is from 21 to 90 mol. % [16]. Souza et al. [17] determined the photoluminescence phenomenon in Ba_y_Ca_1-y_TiO_3_ and attempted to estimate the influence of structural order–disorder, introduced to the material by the substitutions in A sublattice. The increase in light emission intensity with increasing Ca^2+^ ion concentration indicates the introduction of local disorders to the tetragonal lattice and the introduction of small areas with structural features corresponding to the orthorhombic phase.

Ba_y_Ca_1-y_Ti_x_Zr_1-x_O_3_ perovskite ceramics also gain considerable attention for their superior t optical properties as luminescent hosts for one of the most investigated RE ions: praseodymium (Pr^3+^). The contribution of Pr^3+^ to the formation of the microstructure of doped perovskites materials is significant [18]. Apart from the microstructural variations, Pr^3+^ doping can influence the properties of Ba-Ca-Ti-Zr-O ceramics by compositional modifications and lattice defects such as oxygen vacancies formations. It is commonly argued in the literature that the ferroelectric and piezoelectric behavior of Ba-Ca-Ti-Zr-O ceramics is significantly improved by doping Pr^3+^ ions [16,19,20,21]. However, incorporation of Pr^3+^/Pr^4+^ ions is usually aimed at modification of the electronic structure and appearance of new energy levels in the bandgap and, thus, activation of the material toward intense red-light emission [2,10,22,23,24]. Moreover, the mechanoluminescent (ML) phenomenon in the Pr^3+^-doped (Ba,Ca)TiO_3_ diphasic region was reported and investigated [25,26]. The interaction and the intense coupling between the Ba_0.1_Ca_0.9_TiO_3_:Pr^3+^ orthorhombic phosphor phase and the Ba_0.77_Ca_0.23_TiO_3_:Pr^3+^ tetragonal piezoelectric phase acted as determining factors in creating the ML features [27,28].

In the present study, Pr^3+^-doped (Ba,Ca)Ti_x_Zr_(1-x)_O_3_ initial solid solutions with different x (from 0.70 to 1) compositions and Ba:Ca ratio set as 1:1 are investigated. The blue/green photoluminescence in disordered Ba(Ti,Zr)O_3_ solid solutions has been widely studied [11,12,13,14]. There are also several papers dealing with the phenomenon of red photoluminescence in Pr^3+^-doped (Ba,Ca)(Ti,Zr)O_3_ perovskite ceramics sinters [19,20,21,23]. The photoluminescence emission strongly depends on the preparation method and thermal treatment conditions [11,12]. There are a few scientific reports on the synthesis of Ba-Ca-Ti-Zr-O type of ceramics using wet chemical synthesis techniques [29,30,31,32,33,34,35]. In this paper, a polymeric precursor technique, the modified Pechini method, was used to prepare the (Ba,Ca)(Ti,Zr)O_3_ powders. In the investigated ceramics, a few effects determining the occurrence and intensity of the photoluminescence phenomenon are highly probable. The first one is a strong dependence of the luminescence on the structural order–disorder and the changes in the lattice symmetry originated because of ions substitution (mainly in the B sublattice). The second effect is obviously the introduction of Ca^2+^ ions into the A sublattice, leading to a diphasic structure with a separate Ca^2+^-rich phosphor phase. The final factor constitutes the doping with Pr^3+^ ions, which results in the formation of additional luminescence centers. The applications of these factors allowed us to successfully obtain multifunctional material exhibiting a strong photoluminescent effect. It would be of scientific interest to know how the luminescence behavior of Pr^3+^ changes through the Ba-Ca-Ti-Zr-O solid solution system while having different chemical compositions because of Zr^4+^ substitution.

## 2. Materials and Methods

**Figure 1:**
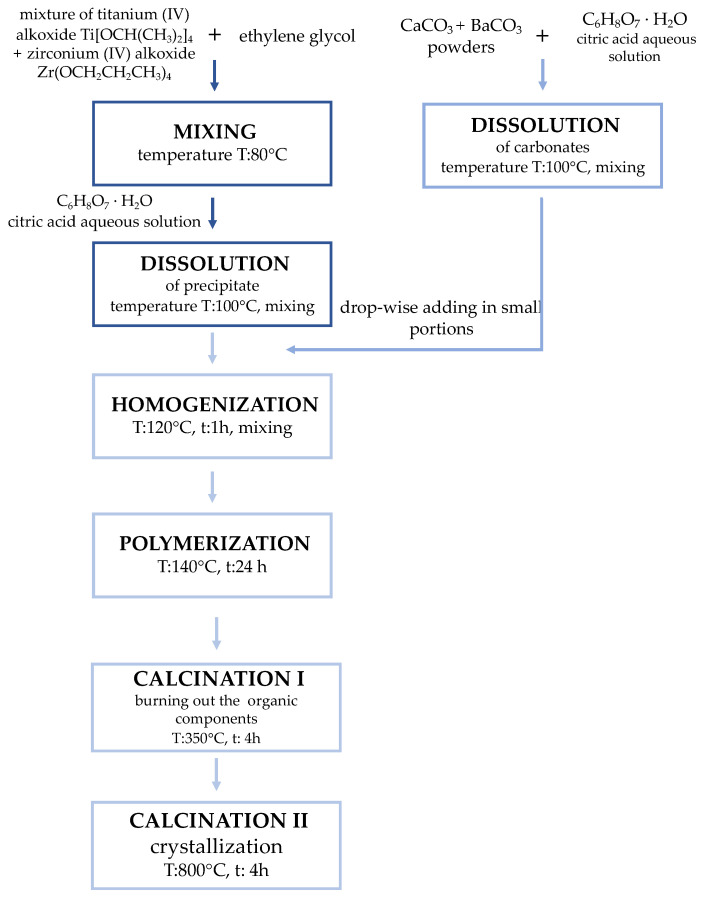
Scheme of obtaining (Ba,Ca)Ti_x_Zr_(1-x)_O_3_ nanopowders by Pechini method.

(Ba,Ca)Ti_x_Zr_(1-x)_O_3_:Pr^3+^, 0.7 ≤ x ≤ 1 with 0.05 step and Ba:Ca ratio set as 1:1 (hereinafter referred to as BCTZ and BCTZ:Pr^3+^ for simplicity) powders were prepared using the Pechini polymeric precursor method. Commercially available titanium (IV) isopropoxide Ti[OCH(CH_3_)_2_]_4_ (Sigma-Aldrich, Saint-Luis, MO, USA), zirconium (IV) propoxide (Zr(OCH_2_CH_2_CH_3_)_4_ 70 wt.% in 1-propanol) (Sigma-Aldrich, Saint-Luis, MO, USA), barium carbonate BaCO_3_, and calcium carbonate CaCO_3_ powders, ethylene glycol C_2_H_6_O_2_, a saturated aqueous solution of citric acid C_6_H_8_O_7_, and rare earth elements in a form of praseodymium (III,IV) oxide Pr_6_O_11_ (each produced by Avantor, Gliwice, Poland) were used as reagents. The selected molar ratio of the sum of cations (Ba^2+^, Ca^2+^, Ti^4+^, Zr^4+^) to citric acid was fixed as 1:2, and the molar ratio of citric acid to ethylene glycol was 1:4. The applied technology chart box is presented in Figure 1.

At the first step of the experiment, the synthesis of the solid solutions of (Ba_,_Ca)Ti_x_Zr_(1-x)_O_3_ (0.7 ≤ x ≤ 1 with 0.05 step) powders were conducted according to the established previously procedure. The resulting polymer resin was dried overnight and then transferred to quarts crucible and heated at 350 °C for 4 h, to decompose the resin and burn out the organic compounds. The solidified lumps were ground to fine powders in a mortar. The optimized temperature of the actual calcination process was as low as 800 °C for 4 h, which allowed the BCTZO perovskite phase formation and crystallization confirmed by X-ray diffraction analysis. At the second stage of the study, the calcined powders were mixed with appropriate weights of praseodymium oxide. The number of praseodymium ions was determined as 0.1 mol. % to the sum of cations of crystallographic sublattice A (Ba^2+^,Ca^2+^), according to a stoichiometric composition (Ba,Ca)_0.999_Pr_0.001_Ti_x_Zr_(1-x)_O_3_. The mixtures of BCTZ:Pr^3+^ powders were thoroughly blended in a mortar, with the addition of polyvinyl alcohol (PVA) binder, and compacted into pellets of 10 mm diameter and 2 mm thickness, using a uniaxial pressure of 50 MPa and subsequently cold-isostatic pressure of 200 MPa. The experimental parameters, amount of Zr^4+^ ion addition, and Pr^3+^ ion concentration were determined based on the author’s preliminary studies.

The final conventional sintering of the BCTZ:Pr^3+^ green bodies was performed at 1450 °C for 4 h at a heating rate of 5 °C/min.

The density of the sintered pellets was measured according to Archimedes’ method. A PANalytical X’Pert Pro (Malvern Panalytical, Malvern, UK) multifunctional diffractometer was used to characterize the structure of the obtained samples using Cu Ka radiation in the range between 20° and 90°. The microstructural observations of the BCTZ:Pr^3+^ materials were performed on sintered pellets using a Nova NANOSem 200 (FEI Company, Hilsboro, OR, USA). Prior to the microstructural investigations, the samples were initially hand- and later machine polished using Diamond Pads (Struers, Cleveland, OH, USA) and MD Polishing Cloth (Struers, Cleveland, OH, USA), with 1 µm diamond paste, to obtain a mirror-like surface. Microstructures of the BCTZ:Pr^3+^ composites were examined on polished and thermally etched (1300 °C, 0.5 h) specimens.

Silver electrodes were coated on the top and bottom surfaces of the specimens for the investigations of the electrical properties. Dielectric properties measurements were conducted using a Quadtech 7600 Plus Precision LCR Meter (Foothill Ranch, CA, USA). To evaluate the photoluminescence properties, the luminescence spectra were recorded at room temperature on spectrofluorometer Edinburgh Instruments FS5 (Edinburgh Instruments, Livingston, UK).

## 3. Results and Discussion

**Figure 2:**
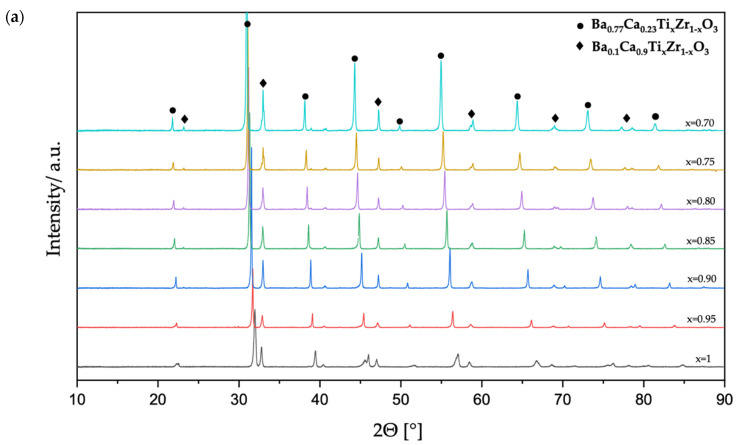

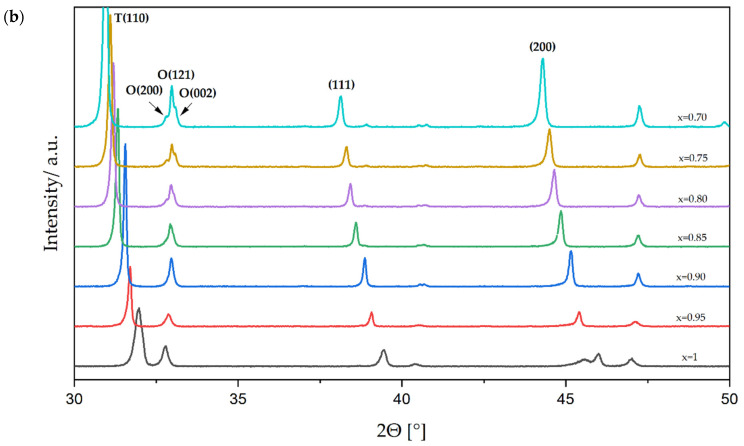
(**a**) The XRD patterns of Pr^3+^-doped (Ba,Ca)Ti_x_Zr_(1-x)_O_3_ ceramics in a 2Θ range of 10–90°; (**b**) refined XRD patterns in a 2Θ range of 30–50°.

The structure of the investigated specimens sintered at 1450 °C for 4 h was determined by X-ray diffraction. The XRD patterns of Pr^3+^-doped BCTZ components are presented in Figure 2. As can be seen, all investigated materials show a pure perovskite-like structure. The XRD analysis revealed that all samples are in fact diphasic and coexistence of orthorhombic Ba_0.1_Ca_0.9_Ti_x_Zr_(1-x)_O_3_:Pr^3+^, and tetragonal–pseudocubic Ba_0.77_Ca_0.23_Ti_x_Zr_(1-x)_O_3_:Pr^3+^ solid solutions was observed. No extra peaks resulting from impurity or byproducts were detected, suggesting that Zr^4+^ and Pr^3+^ ions incorporated into both phases substituting the Ti^4+^ ions in B sublattice and the Ba^2+^/Ca^2+^ ions in A sublattice, respectively [10,25]. Moreover, the position of the strongest peak originated from tetragonal–pseudocubic phase T(110) shifts evidently toward the lower-angle side with increasing Zr^4+^ content. Similar slight position changes can be observed for other peaks assigned to the tetragonal–pseudocubic phase as well. This indicates lattice expansion because of larger Zr^4+^ ions substitution for smaller Ti^4+^ ions, which could be confirmed by the Williamson–Hall calculations [36]. The Rietveld analysis indicated that the Ba_0.77_Ca_0.23_Ti_x_Zr_(1-x)_O_3_:Pr^3+^ phase has a tetragonal structure for x = 1, which is indicated by the split of the (200) peak around 2Θ~45°. For 0.70 ≤ x < 1, the structure turns into pseudocubic. When it comes to Ba_0.1_Ca_0.9_Ti_x_Zr_(1-x)_O_3_:Pr^3+^ phase, with increasing the Zr^4+^ content, the orthorhombic peaks of (200), (121), and (002) separates clearly, which confirm high orthogonality of the mentioned phase. The position of the orthorhombic (121) peak shifts evidently toward the higher-angle side with increasing Zr^4+^ from 0 to 0.10 mol. %. The peak shifts indicate a shrinkage of the lattice of the orthorhombic Ca^2+^-rich phase, despite the substitution of larger Zr^4+^ ions. This is possibly due to the appearance of nonstoichiometry in the A sublattice and the substitution of some Ba^2+^ ions by smaller Ca^2+^ ions. For x < 0.9, the peak shift is negligible and results only from peak splitting.

The calcination temperature observed in this study is even ~300–500 °C less than that reported for the conventional solid-state reaction process [37]. Consequently, we can state that we had developed much more energy-efficient sintering processes, considering the techno-economic analysis of Pechini’s method implementation. This is very important in the ceramic industry in which energy-intensive and decarbonizing ceramic manufacturing have necessitated more efficient manufacturing, particularly through the introduction of modern preparation technologies [38].

The reduced calcination and sintering temperature obtained in this case are possibly due to the high reactivity rate of nano-precursor powders resulting from the modified Pechini synthesis process. The average crystalline sizes were estimated using the Debye–Scherrer relation between the size of crystallites and the line broadening at half of the maximum intensity of Bragg’s peaks. This method allows us to obtain crystallite sizes in the obtained powders as small as 12–15 nm, whereas even in the experiment by Praveen et al. [39], the crystallite size was only 30 nm. The crystallite sizes, estimated in the same way in the BCTZ:Pr^3+^ sinters, were between 78 nm and 145 nm, for the Ca-rich phase, and between 75 nm and 181 nm, for the Ba-rich phase, decreasing gradually with increasing Zr^4+^.

**Figure 3:**
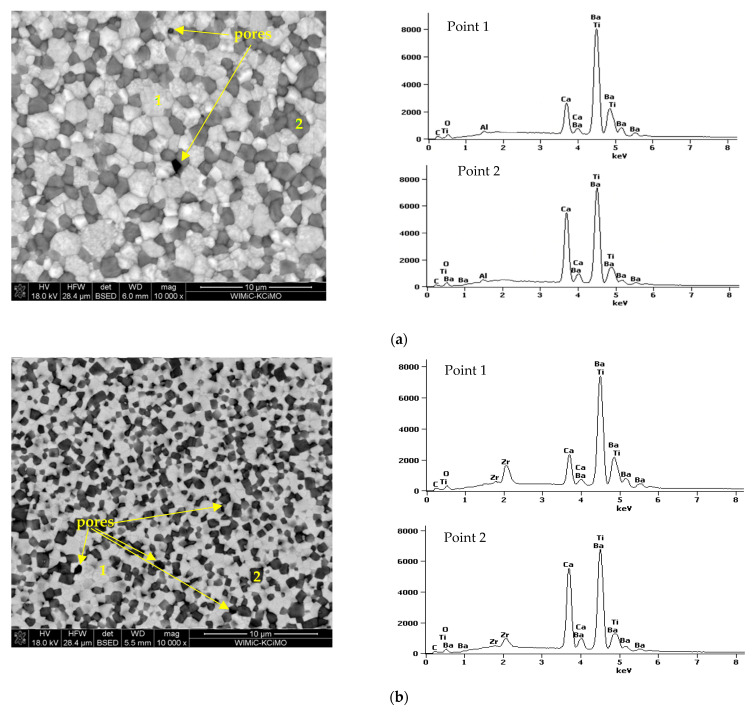

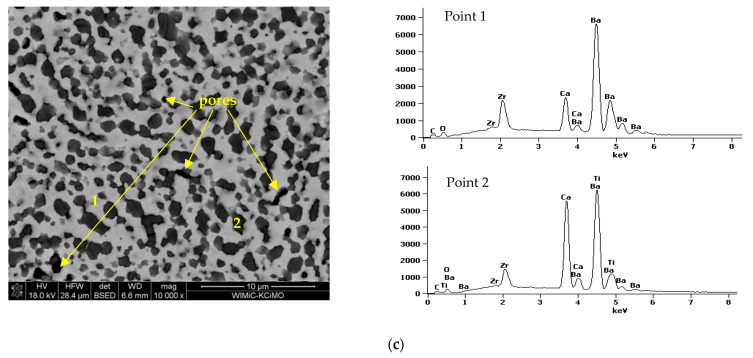
SEM images of the polished and thermally etched microstructures of the selected BCTZ:Pr^3+^ diphase ceramics obtained by Pechini method and sintered at 1450 °C (on the left) and the corresponding EDS scan spectra of the two marked points (on the right), respectively (**a**) x = 1 (**b**) x = 0.9 and (**c**) x = 0.8.

The typical examples of SEM micrographs of the microstructure observed on polished and thermally etched surfaces of BCTZ:Pr^3+^ samples are shown in Figure 3. All specimens were characterized by well-dense, uniform microstructures, and only single pores were observed. The determined relative density for all compositions was more than 95%. The SEM observations, in line with the XRD analysis, confirmed that the ceramics were definitely diphasic and consisted of light Ba_0.77_Ca_0.23_Ti_x_Zr_(1-x)_O_3_:Pr^3^ grains (Ba-rich phase with bright contrast) and dark Ba_0.1_Ca_0.9_Ti_x_Zr_(1-x)_O_3_:Pr^3+^ phosphor grains (Ca-rich phase with dark contrast), which were clearly verified by EDS point scans also presented in Figure 3. The pictures show numerous shadows, suggesting the presence of dark Ca-rich phase particles shallowly below the surface of the specimen—no intermediate phase was determined. The average grain size was estimated to be ~1–2 μm for BCTZ:Pr^3+^. As the Zr^4+^ ions incorporate into the perovskite structure, the grain size decreases, and the greater content of the small grains below 1 μm is evident. Based on the literature results, density and grain size strongly influence the PL properties of rare-earth-element activators, especially when grain size did not exceed a certain value [40]. Zhang et al. [22,23] established that, in the Pr^3+^-doped Ba-Ca-Ti-Zr-O system, the optimal value of grain sizes should not exceed 8 μm, which corresponds well with the presented microstructures in this study.

The analysis of surface structure revealed perfect grain arrangement in the ceramics obtained by the investigated method and homogenous grain distribution throughout the microstructure (so-called piezoelectric/phosphor/piezoelectric sandwich structure) [28]. Moreover, in the SEM images, it can be clearly observed that the grains in the phosphor Ca-rich phase are tightly surrounded by the second-phase particles, owing to the self-assembled sandwich architecture, and very intense interaction between them could occur. The strong diphasic coupling occurred on the nano- and micrometer scales. Strong interaction between both phases in self-submitted sandwich structure obtained using solid-state reactions was also confirmed by Zhang et al. [28].

**Figure 4:**
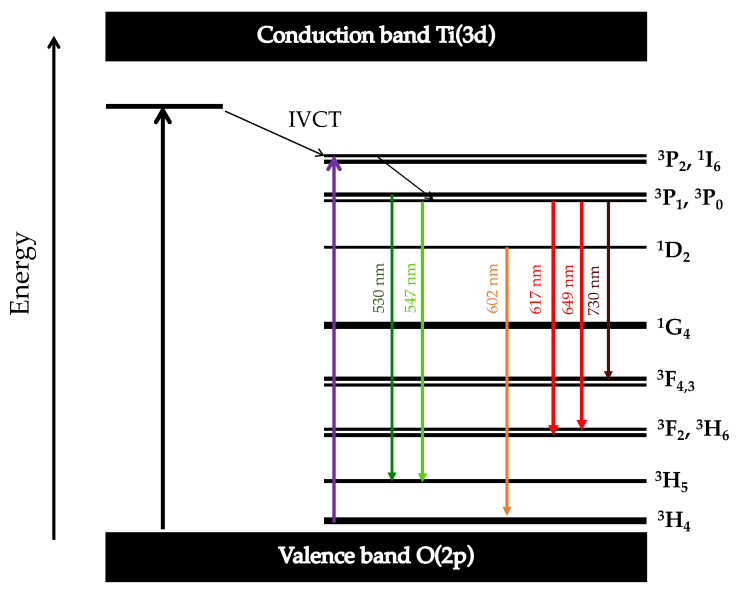
Energy level diagram for the Pr^3+^ ions [19,23,24].

It is assumed that the Pr^3+^ ion possesses its own intrinsic luminescent properties and—once incorporated into host A sublattice—provides greenish-blue and reddish-orange luminescence centers. Figure 4 illustrates the energy level diagram of Pr^3+^ ions. According to it and following the selection rules, the electrons from the ^3^H_4_ ground states are excited to the ^3^P_i_ (I = 0, 1) and ^3^P_2_ high level and then radiationlessly de-excited to the ^3^P_i_ (I = 0, 1) and ^1^D_2_ states. The emission peaks are credited to the typical f–f transition [41]. The greenish-blue emission is located at 530 nm and 547 nm, which corresponds to the ^3^P_1_-^3^H_5_ and ^3^P_0_-^3^H_5_, respectively. The reddish-orange color emission exhibits three strong peaks centered at 602 nm, 617 nm, and 649 nm, which are associated with the recombination between ^1^D_2_-^3^H_4,_^3^P_0_-^3^H_6_, and ^3^P_0_-^3^F_2_, respectively [19,23]. However, the presented approach in this paper is simplistic, and the interactions between Pr^3+^ dopant and crystalline and electronic host structures are not negligible and can ultimately determine the luminescent characteristics of the material [10,42].

**Figure 5:**
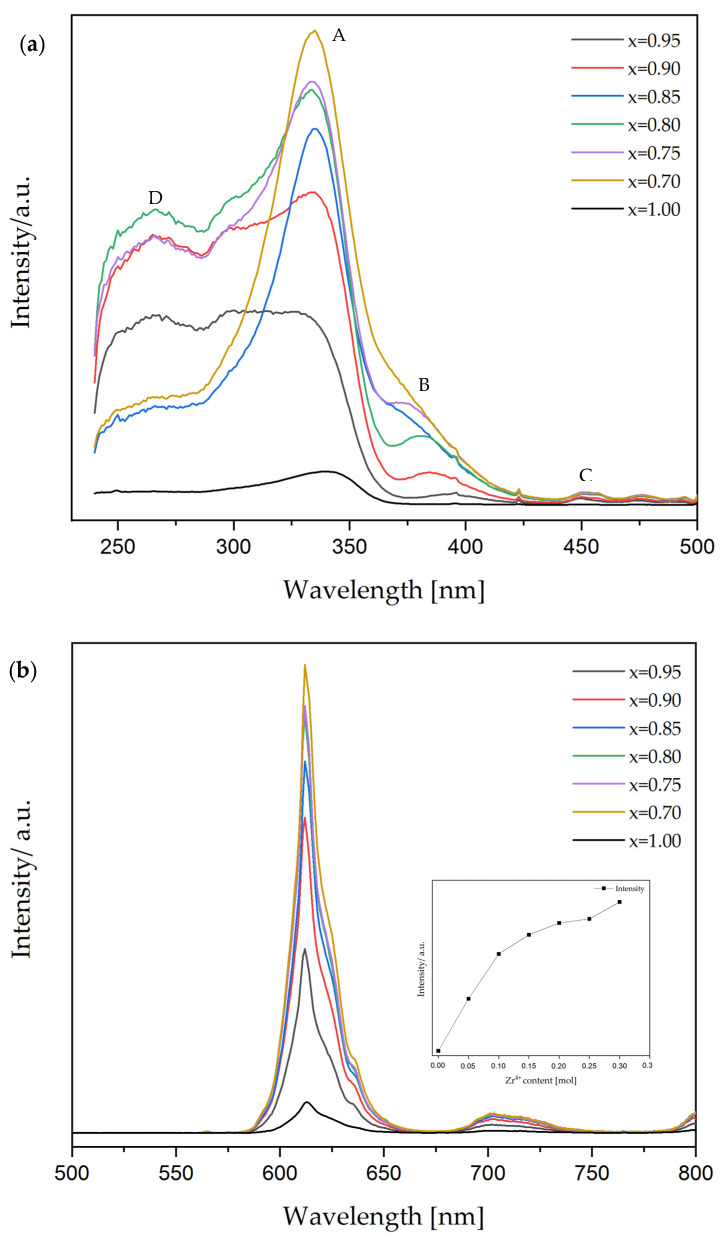
(**a**) Excitation spectra and (**b**) emission spectra of BCTZ:Pr^3+^ ceramics at room temperature. The insert is the emission intensity changes with the Zr^4+^ substitution.

**Figure 6:**
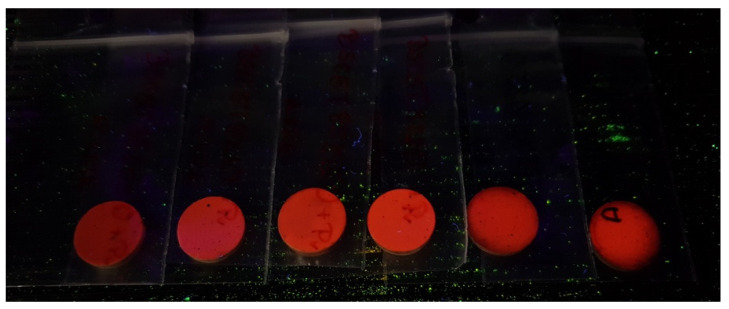
Red-light emission of the investigated samples visible by the naked eye.

Figure 5a shows the photoluminescence excitation spectra of BCTZ:Pr^3+^, 0.7 ≤ x ≤ 1 ceramics monitored at 612 nm wavelength. The observed bands were labeled with the letters A, B, C, and D. According to the literature [10,43,44], A band is ascribed to the valence to conduction band transition of the host [O (2p)–Ti (3d)], B band resulted from the Pr^3+^ to metal charge transfer state (also known as intervalence charge transfer IVCT state), and C band is assigned to the ^3^H_4_ to ^3^P_i_ (I = 0, 1, 2) transitions (f–f transition), respectively. Moreover, the D band demonstrated the lowest field component of the 4f5d state of Pr^3+^ [44,45]. Broadbands were observed, which became sharper with Zr^4+^ content, increasing in the UV range from 230 nm to 475 nm. The absorption peaks reached their maxima at the same wavelength around 340 nm, and no significant shift was detected. Figure 5b presents the photoluminescence emission of Pr^3+^-doped BCTZO ceramics with different Zr^4+^ content excited at 340 nm wavelength at room temperature. The emission spectra patterns are identical to the characteristics of emission lines of Pr^3+^ ions. No visible shift was observed, and the narrow emission bands for all samples were centered. peaking at 612 nm, which corresponds to ^1^D_2_-^3^H_4_ recombination [44]. The photoluminescence intensity gradually increased when the Zr^4+^ was incorporated in the structure, achieving the highest emission for the BCTZ:Pr^3+^ with x = 0.7. The red-light emission of the investigated samples is shown in Figure 6.

The substitution of Ti^4+^ by Zr^4+^ in Pr^3+^-doped BCTZ ceramics is supposed to impact the optical properties of the material. The enhancement of the photoluminescence intensity with the increasing Zr^4+^ content can be caused by a few factors. An important factor might be a change in local lattice symmetry induced by the Zr^4+^ ions substitution for Ti^4+^ ions. It was stated [10] that lattice variations have a small effect on the f–f transition (C band). However, it strongly influenced the conduction band and the host absorption (A band). Moreover, 4f5d energy levels of Pr^3+^ are highly sensitive to the changes of the crystalline environment and local field around Pr^3+^. Consequently, the outermost 5s and 5p electrons shells are shifted, and the 5d energy level changed its position, allowing stronger interactions between the 4f energy level and the host, thus stronger 4f–4f emission. The second factor leading to improvement of PL intensity is closely related to the high stability of Zr^4+^ ions (higher than Ti^4+^ ions) [10]. It is well known [42] that the Pr^3+^ ion located in the BCTZ structure has the ability to donate an electron to form the Pr^4+^. The released electron is taken over by Ti^4+^, resulting in its reduction to Ti^3+^. This process involves the creation of a large number of positively and negatively charged defects, which have great effects on luminescence quenching [10,46]. For example, they can reabsorb the energy from the red-light emission or act as competitive traps for the excited electrons in the conduction band [47,48]. The phenomena affect the weakening of the emission intensity. The very stable Zr^4+^ ion is unwilling to accept an electron and changes its oxidation state to Zr^3+^. Therefore, a higher number of Zr^4+^ ions contribute to an increase in the number of Pr^3+^ ions (not Pr^4+^) and a decrease in the concentration of defects in the structure. Lopez-Pacheco et al. [42] stated that structural-related modifications occurring with Zr^4+^ substitution change the interaction between host and Pr^3+^ (f–f transition), resulting in the favorable and efficient energy transfer between host and Pr^3+^.

The greenish-blue emission was absent in the investigated Pr^3+^-doped BCTZ ceramics. The origin of dominant red emission in Pr^3+^-doped ceramics is a matter of ongoing discussion. Reut and Ryskin [49] and Boutinaud [50] proposed a metal-to-metal charge transfer (MMCT–IVCT) model with Pr^4+^-O^2—^Ti^3+^ excited state. Excited f electron of Pr^3+^ undergoes relaxation to ^1^D_2_, simultaneously quenching the emission from ^3^P_0_ and ^3^P_1_ states. Barandarian et al. [51] presented the conception of O^2—^Ti^4+^ ligand-to-metal charge transfer (LMCT), which could be responsible for nonradiative deexcitation from ^3^P_0_ to ^1^D_2_ and, therefore, dominant red luminescence. Another idea is that both charge transfer states occur in the titanates at the same time and are influenced by host structure and composition [42]. Sun et al. [43] indicated that the overlap between the conduction band of the host and the 4f5d levels of the Pr^3+^ is responsible for transferring the excited electron from 4f5d, through the conduction band and nonradiative deexcitation to the ^1^D_2_ level. According to the author, the ^3^P_i_ (i = 0, 1) emission might be fully quenching by cross-relaxation through the 4f5d levels and MMCT.

**Figure 7:**
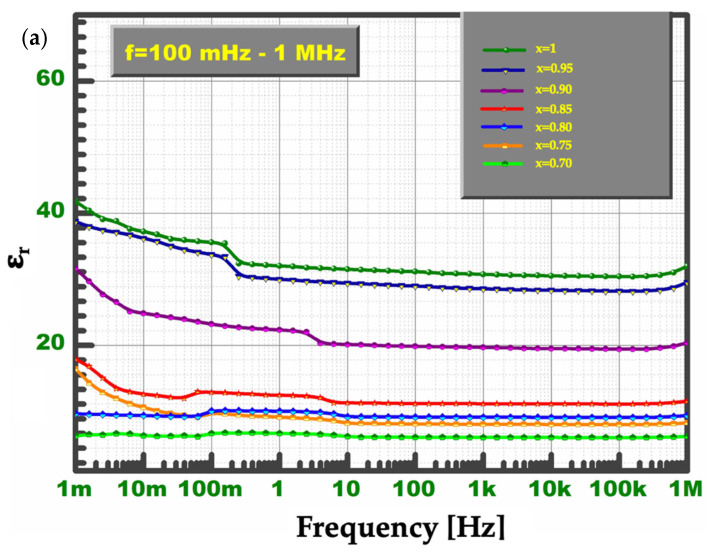

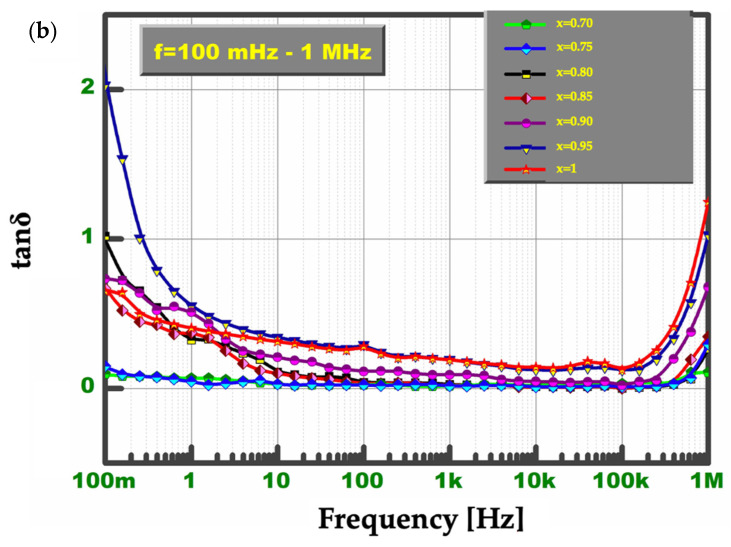
Frequency-dependent dielectric properties of investigated BCTZ:Pr^3+^ ceramics: (**a**) ε_r_ and (**b**) tanδ.

Presented in Figure 7 dielectric constant (e_r_) and dielectric loss (tanδ) frequency dependence for all BCZT:Pr^3+^ ceramics were measured at room temperature. As can be seen in Figure 7a, the e_r_ values of the investigated ceramics are almost consistent in the frequency range from 10 mHz to 1 MHz. Distinct increases in this parameter’s values were recorded with the decrease in frequency. Such a higher dielectric constant level in the low-frequency range may originate from the response of various polarization mechanisms in diphasic material. Moreover, the dielectric constant value noticeably decreases with the increase in Zr^4+^ content. According to Figure 7b, the dielectric loss values of all BCZT:Pr^3+^ ceramics show a low level (tanδ < 0.4) and remain almost unchanged in the wide frequency range from 10 Hz to 100 kHz. However, the tanδ values slightly increase with the decrease in frequency, similar to the dielectric constant, but markedly change to higher values in the high-frequency range. Substitution of larger Zr^4+^ for smaller Ti^4+^ ions induces compressive stresses on the adjacent lattice, weakens the bonding force between B-site cations and oxygen ions, and destabilizes the tetragonal structure, resulting in a formation of pseudocubic phase. In addition, some compositional disorder may occur, leading to the diffusive phase transition phenomena [8]. Moreover, the substitution of Zr^4+^ may induce the rearrangement of oxygen ions in oxygen octahedrons, affecting the reduction in the space of Ti^4+^ ion vibrations. Hence, the suppression of spontaneous polarization of Ti^4+^ occurs [6]. These factors result in a decrease in the dielectric constant value and tanδ and a slight deterioration of the dielectric properties with the increase in Zr^4+^ content.

## 4. Conclusions

For the first time, photoluminescence was recorded in Pr^3+^-doped BCTZ perovskite diphasic ceramics obtained by the modified Pechini method, and the strong red-light emission can be observed with the naked eye. The application of the method from the wet chemistry group allowed obtaining the material characterized by a homogeneous, fine, and dense microstructure at a significantly low temperature (calcination at 800 °C, sintering at 1450 °C).

The optical properties were significantly improved with the Ti^4+^ substitution by stable Zr^4+^. The incorporation of Zr^4+^ ions induces local lattice variation and decreases the concentration of photoluminescent-quenching defects in the structure, which leads to the enhancement of photoluminescence effects. Electrical properties investigations show that Pr^3+^-doped BCTZ ceramics possess stable dielectric properties with low tanδ values. As a result, the Pr^3+^-doped BCTZO ceramics are promising candidates for environmentally friendly and versatile materials by combining good electrical and photoluminescence properties, with a prognosis for the manifestation of multifunctional effects.

## Data Availability

The data presented in this study are available on request from the corresponding authors.
